# A very rare case of extraskeletal Ewing sarcoma of the duodenum presenting as gastrointestinal hemorrhage

**DOI:** 10.1055/a-2085-0337

**Published:** 2023-06-12

**Authors:** Zilong Zhang, Xin He, Songqi Wen, Xin Jin, Ding Xiao, Jian You

**Affiliations:** Department of Hepatobiliary-Pancreatic and Hernia Surgery, Wuhan Fourth Hospital, Puai Hospital, Tongji Medical College, Huazhong University of Science and Technology, Wuhan, China

A 51-year-old man presented to our hospital with hematochezia, melena, palpitations, and lightheadedness for 1 week. The patient was found to have anemia with a hemoglobin concentration of 73 g/L and a peripheral blood smear suggestive of hypochromic microcytic anemia. To confirm upper gastrointestinal bleeding, an esophagogastroduodenoscopy was performed, revealing a protruding lesion with surface ulceration and active bleeding at the inferior flexure of the duodenum ( Fig. 1 a ). The lesion was located on top of a submucosal elevation of approximately 3 × 2.5 cm in size ( Fig. 1 b, c ). He received endoscopic hemostasis, including epinephrine injection, argon plasma coagulation, and hemoclipping ( Fig. 1 d , Video 1 ). The biopsy was suggestive of small cell carcinoma. To obtain more details for the optimal surgical approach, abdominal computed tomography (CT) and magnetic resonance cholangiopancreatography (MRCP) showed a target-like lesion with eccentric wall thickening at the inferior flexure of the duodenum, measuring approximately 2.8 × 2.4 cm in diameter ( Fig. 2 a–c ).

**Fig. 1:**
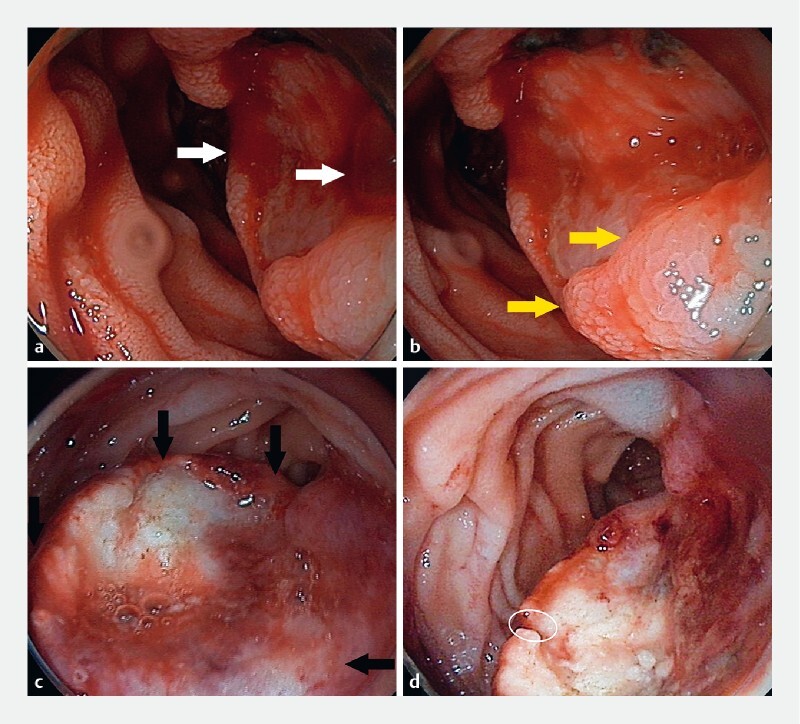
Esophagogastroduodenoscopy images. **a** A tumor at the second part of the duodenum causing surface ulceration and active bleeding (arrow). **b** Unsmooth surface of the tumor with the appearance of erosion or ulcer (arrow). **c** Endoscopic narrow-band imaging view of the lesion. **d** Biopsy sampling from tumor margin (circle).

**Video 1** Esophagogastroduodenoscopy is performed for ulcerative tumor, including epinephrine injection, argon plasma coagulation, hemoclipping, and biopsy. The tumor was completely resected by laparoscopic pancreaticoduodenectomy and was confirmed as extraskeletal Ewing sarcoma of the duodenum on histology.


**Fig. 2:**
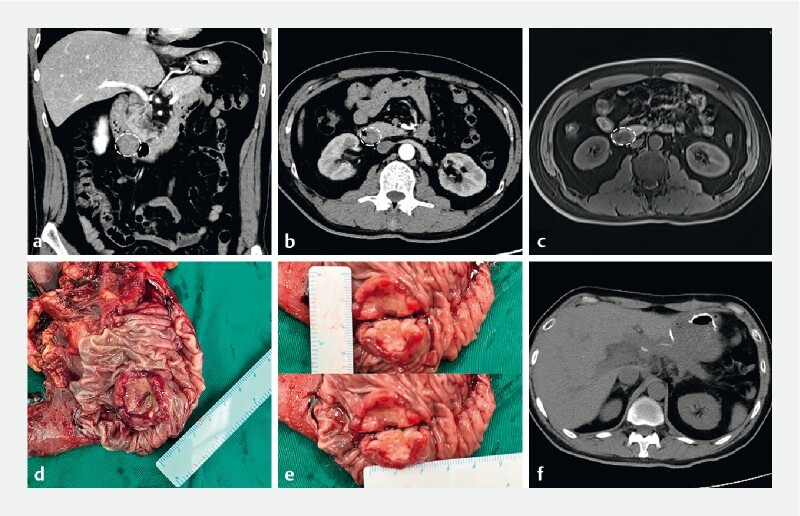
Imaging studies and gross appearance of a tumor. **a** Abdominal computed tomography (CT) showed – on coronal view – a target-like lesion with eccentric wall thickening at the inferior flexure of the duodenum (circle). **b** Axial view showed a sausage-shaped lesion (circle). **c** Magnetic resonance cholangiopancreatography showed lesion with localized thickening of the anterior wall of the bowel, measuring approximately 2.8 × 2.4 cm in diameter (circle). **d** Gross appearance showed an ulcerative tumor at the inferior flexure of the duodenum, measuring 3 × 2.5 cm in diameter. **e** Tumor profile showed the tumor had a gray-white cup-like shape appearance with solid and cauliflower-like areas. **f** Follow-up CT at 2 months postsurgery showing no sign of tumor recurrence or metastasis.

The patient underwent a laparoscopic pancreaticoduodenectomy for a duodenal carcinoma. Gross examination was notable for an ulcerative tumor of approximately 3 × 3 × 2.5 cm in diameter at the inferior flexure of the duodenum, which had gray-white cup-like shape appearance with solid and cauliflower-like areas ( Fig. 2 d, e ; Video 1 ). Histological and immunohistochemical examination revealed poorly differentiated extraskeletal Ewing sarcoma of the duodenum, which was positive for CD99/FLI-1/INI-1/Ki67(60%)/NKX2.2 but negative for CK-P/LCA/S100/CD56/WT-1/desmin ( Fig. 3 ). Upon genetic testing, a fusion of the *EWSR1* gene on chromosome 22q12 was found with the gene encoding the transcription factor ERG (exon 8 of *EWSR1* fused to exon 9 of ERG) and a wild-type *KRAS* / *NRAS* / *BRAF.* Postoperatively, the patient was treated with interval-compressed VDC/IE chemotherapy. Two months after surgery, the CT showed no sign of tumor recurrence or enlarged lymph nodes ( Fig. 2 f ).

**Fig. 3:**
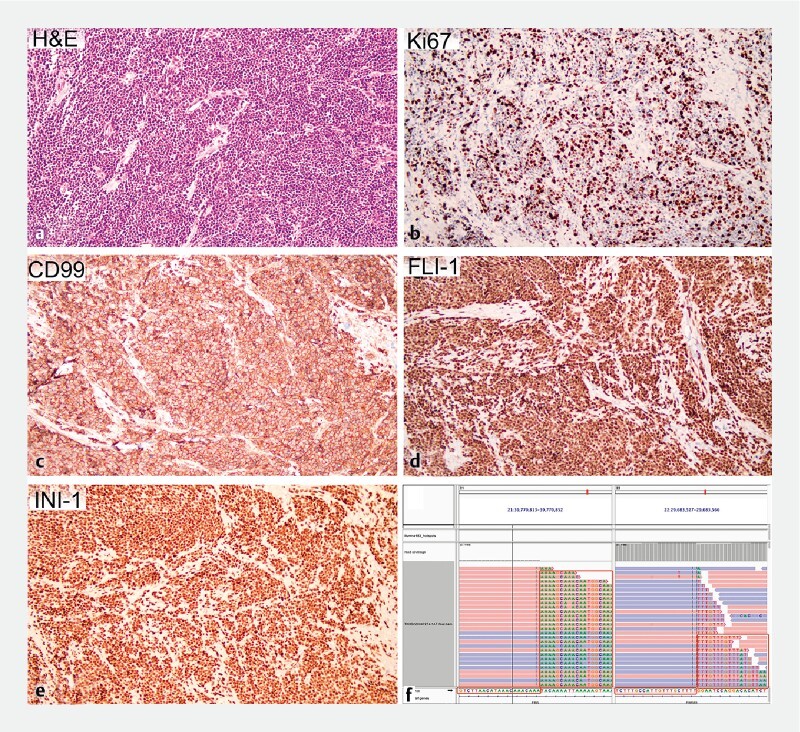
Tumor histology consistent with poorly differentiated small round cell tumor. **a** Hematoxylin and eosin, × 200, with no lymphovascular invasion and a negative vertical margin. **b** Immunohistochemically, the Ki-67 (× 200) index of small round cell tumor cells was 60 %. **c–e** Tumor positive for CD99 (× 200) and FIL-1 (× 200), INI-1 (× 200). **f** Genetic testing showed *EWSR1– ERG* fusion.

Extraskeletal Ewing sarcoma of the duodenum is an extremely rare tumor with a poor prognosis 1 . The diagnosis of extraskeletal Ewing sarcoma of the duodenum is complicated because of the lack of specific clinical symptoms and imaging findings 2 . There is no treatment guideline for this type of cancer. Our case suggests that patients presenting with recurrent hematochezia, weight loss, an abdominal mass, and anemia should receive esophagogastroduodenoscopy for diagnosis. It seems that surgical resection and adjuvant chemotherapy may contribute to a relatively good survival outcome for patients with extraskeletal Ewing sarcoma of the duodenum.

Endoscopy_UCTN_Code_CCL_1AB_2AZ_3AB

## Notes

Endoscopy E-Videos

https://eref.thieme.de/e-videos
*E-Videos* is an open access online section of the journal *Endoscopy* , reporting on interesting cases and new techniques in gastroenterological endoscopy. All papers include a high-quality video and are published with a Creative Commons CC-BY license. Endoscopy E-Videos qualify for HINARI discounts and waivers and eligibility is automatically checked during the submission process. We grant 100% waivers to articles whose corresponding authors are based in Group A countries and 50% waivers to those who are based in Group B countries as classified by Research4Life (see: https://www.research4life.org/access/eligibility/ ). This section has its own submission website at https://mc.manuscriptcentral.com/e-videos
